# Measuring light exposure in nursing home residents with dementia: facility-level measures vs wearable sensor

**DOI:** 10.1093/geroni/igaf122.3289

**Published:** 2025-12-31

**Authors:** Ying-Ling Jao, Yo-Jen Liao, Diane Berish, Julian Wang, Margaret Calkins, Yun-Han Kuan, Light Ndurue, Marie Boltz

**Affiliations:** The Pennsylvania State University, University Park, Pennsylvania, United States; IDEAS Institute, Cleveland Heights, Ohio, United States; The Pennsylvania State University, University Park, Pennsylvania, United States; The Pennsylvania State University, University Park, Pennsylvania, United States; IDEAS Institute, Cleveland Heights, Ohio, United States; Far Eastern Memorial Hospital, New Taipei City, Taipei, Taiwan (Republic of China); The Pennsylvania State University, University Park, Pennsylvania, United States; The Pennsylvania State University, University Park, Pennsylvania, United States

## Abstract

Adequate lighting exposure has positive impacts on circadian health, mood, and behavioral symptoms in people with dementia. However, nursing home (NH) residents with dementia often experience insufficient circadian lighting exposure. Increasing research has investigated lighting interventions in this population with mixed results. Most intervention studies evaluated outcomes based on facility-level lighting measures without considering the individual’s actual light exposure. However, light exposures can vary widely between residents, depending on room location, personal behavior, and daily routine. Wearable light sensors allow us to quantify the dosage each individual receives more precisely. This study examined the differences in light intensity (lux) between facility-level and individual-level measures among NH residents with ADRD. The facility-level measure was conducted twice a week in the bedroom, hallway, and dining room, tailored based on individuals’ routines. Individual lighting exposure was measured using a wearable light sensor, LYS button, worn on residents’ shoulder/collar 24 hours/day continuously for one week. Descriptive statistics and paired t-tests were performed. A total of 29 residents (average age: 87 years; 72.4% female) were recruited from two NHs. The average facility-level lighting intensities from the two NHs were 110, 114, and 85 lux in the bedroom, hallway, and dining room, respectively. In contrast, residents’ individual lighting exposure averaged 268 lux. Paired t-tests revealed significant differences between facility-level manual measures and the individual wearable sensor in the bedroom, hallway, dining room, and the overall facility (p < 0.001). Future research should consider using individual light sensors for a more precise design and evaluation of lighting interventions.

